# Measurement of methotrexate in human cerebrospinal fluid using a chemiluminescence immunoassay intended for serum and plasma matrices

**DOI:** 10.1002/jcla.23661

**Published:** 2020-11-22

**Authors:** Naoki Yoshikawa, Ai Yamada, Tsubasa Yokota, Hiroshi Moritake, Yasutoshi Hirabara, Ryuji Ikeda

**Affiliations:** ^1^ Department of Pharmacy University of Miyazaki Hospital Miyazaki Japan; ^2^ Division of Pediatrics Faculty of Medicine University of Miyazaki Miyazaki Japan

**Keywords:** cerebrospinal fluid, chemiluminescence immunoassay, high‐performance liquid chromatography, methotrexate, Passing‐Bablok

## Abstract

**Background:**

The concentration of MTX in blood is often measured quickly and easily by immunoassays. Thus, immunoassays may facilitate the easy determination of the concentration of MTX in the cerebrospinal fluid (CSF). In this study, we measured methotrexate (MTX) concentrations in the CSF using a high‐performance liquid chromatography (HPLC) method intended for analyzing CSF matrices and a chemiluminescence immunoassay (CLIA) method intended for assessing serum and plasma matrices and verified the differences in the results of the two methods.

**Methods:**

HPLC analysis for MTX in the CSF was performed using a Prominence UFLC system with a C18 column. The HPLC method was validated in accordance with the 2018 FDA guideline. The CLIA method was performed using an ARCHITECT i1000SR system intended for serum and plasma matrices. A total of 47 CSF samples (14 clinical and 33 spiked specimens) were analyzed using the two methods.

**Results:**

The HPLC method passed the validation criteria. The concentration of MTX in the same sample, determined using the HPLC and CLIA methods, differed proportionally; the percent difference in the concentrations averaged −23.0% (95% confidence interval: −36.9% to −9.1%) as revealed by the Bland‐Altman plot. The relationship between the measured values, evaluated using the Passing‐Bablok regression, was as follows: *HPLC* = 1.205 × *CLIA* – 0.024.

**Conclusion:**

The equation deduced in this study can be used to correct the concentration of MTX measured using the CLIA method.

## Graphical abstract

MTX concentrations in the CSF were measured using a HPLC method intended for analyzing CSF matrices and a CLIA method intended for assessing serum and plasma matrices, and the differences in the results of the two methods were verified. The concentration of MTX in the same sample, determined using the HPLC and CLIA methods, differed proportionally.

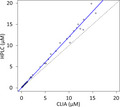

## 1 INTRODUCTION

Methotrexate (MTX) is a cytotoxic drug of the antifolate type, which is used to treat certain types of hematological cancers, solid tumors, and rheumatoid arthritis. MTX, at high doses, is used clinically as a cytotoxic drug for the treatment of solid tumors and leukemia.^1^, ^2^ Regular monitoring of MTX concentrations in the serum allows early detection of abnormal clearance so that measures, such as adjustment of the dose of leucovorin and enhancement of hydration, can be opted to prevent excessive toxicity.^3^, ^4^ If extracorporeal excretion is slower than the standard, enhancement of the normal cell rescue by the administration of leucovorin should be considered. Methotrexate is administered intrathecally as a measure against the recurrence of acute lymphocytic leukemia in the central nervous system to improve the therapeutic results. The intraventricular administration of anticancer drugs has the advantage of maintaining high drug concentrations in the cerebrospinal fluid (CSF) and minimizing systemic side effects. However, sustained exposure to high concentrations of anticancer drugs is dangerous, warranting measures to avoid it. Methotrexate is cytotoxic at concentrations of more than 1 µM.^5^ It has been reported that although MTX concentrations in the CSF do not exceed 1 µM after intravenous administration, they can be higher immediately after intrathecal administration.^6^ Intraventricular drug clearance is not uniform in all patients. Therefore, to safely administer the anticancer drug by intrathecal injection, evaluation of local pharmacokinetics after administration is required.

The concentration of MTX in blood is often measured by immunoassays. The results can be obtained easily and quickly using this method, thereby greatly contributing to the management of delayed extracorporeal excretion. For the immunoassay of MTX concentrations in blood, fully automatic measuring devices are widely used, most of which use serum or plasma as the target matrix. Separation analyses methods, such as capillary electrophoresis ^7^ and LC‐MS/MS,^8^ are mainly used to measure MTX concentrations in the CSF. Although the separation analyses methods are highly versatile, they require advanced analytical techniques, unlike the immunoassay methods. Therefore, it is not very feasible to measure the concentration of drugs using separation analyses in daily clinical practice.

The use of immunoassay as an alternative to the separation analyses methods can make the routine determination of the concentration of MTX in the CSF easy. Herein, we focused on the use of chemiluminescence immunoassay (CLIA) for this purpose. The CLIA method is now widely used to measure the concentration of MTX in blood.^9^, ^10^, ^11^ In the present study, we aimed to construct an environment for measuring the concentration of MTX in the CSF using a CLIA method following past reports.^12^, ^13^ We measured the concentration of MTX in CSF using a high‐performance liquid chromatography (HPLC) method, intended for CSF, and a CLIA method, intended for serum and plasma matrices. We also determined the differences in the results obtained using the two methods. We believe that our results will be significant for employing the CLIA method to determine the MTX concentration in the CSF.

## 2 MATERIALS AND METHODS

### 2.1 Materials

Methotrexate and phosphate‐buffered saline (PBS) (pH 7.4) were obtained from Sigma‐Aldrich. 1,3,7‐Trimethyluric acid (137U) was obtained from Cayman Chemical. Acetonitrile (LC‐MS‐grade) was obtained from Merck. HPLC‐grade methanol and water were obtained from FUJIFILM Wako. Acetic acid, sodium acetate, sodium hydroxide (NaOH), and hydrochloric acid (HCl) were obtained from Nacalai Tesque.

### 2.2 Instrumentation

HPLC analysis was performed on a Prominence UFLC system (Shimadzu). The Analyst software (LCsolution, Shimadzu) was used to acquire and process the data. The CLIA method was performed on an ARCHITECT i1000SR system (Abbott).

### 2.3 HPLC conditions

Chromatographic separation was done at 40°C using an InertSustain AQ‐C18 column (3 µm HP, 150 mm × 3 mm; GL Sciences, Tokyo). The mobile phase consisted of sodium acetate buffer (50 mM, pH 3.6):acetonitrile (77:13, v/v). The flow rate was 0.9 mL/min. The detector wavelength was set at 307 nm.

### 2.4 Preparation of stock and working solutions

MTX stock solution (100 mM) and the internal standard (IS) (137U, 10 mM) were prepared in 0.01 M NaOH and HPLC‐grade water, respectively, and stored at −20°C. The concentration of the IS working solution was 100 µM.

### 2.5 Preparation of calibration and quality control samples

For drug‐free CSF, the sample that remained after CSF testing was used. Matrix calibrators were prepared daily by the addition of properly diluted stock solution to drug‐free CSF. A series of calibration samples (0.10, 0.25, 0.50, 0.75, 1.00, 1.25, and 1.50 µM) was prepared. The lower limit of quantitation (LLOQ) (0.10 µM) and three quality control (QC) samples at low (0.20 µM), medium (0.60 µM), and high (1.20 µM) concentrations of MTX were prepared by the addition of appropriately diluted stock solution to drug‐free CSF.

### 2.6 Patient enrollment, sample collection, and preparation

The leftover CSF test samples collected from patients receiving MTX intrathecally were used as clinical specimens. Spiked specimens were prepared by the addition of properly diluted stock solution to drug‐free CSF.

For the solid phase extraction procedure,^14^ the Oasis HLB 1 cc Vac Cartridge (Waters, Milford, MA, USA) was used. A sample of 100 µL of CSF was mixed with 100 µl of 1 M HCl, 50 µl of IS solution, and 700 µl of PBS (‐) and was injected into the cartridge, which was preconditioned with 1 ml of methanol followed by 1 ml of H_2_O. Subsequently, the cartridges were washed with 1 ml of 5% methanol in 0.01 M HCl. Finally, MTX and IS were eluted with 1 ml of methanol. The eluent was collected in conical disposable glass tubes and evaporated under a gentle stream of nitrogen at 45°C. The residue was dissolved in 100 µl of PBS and centrifuged (14 000 × *g*, 4°C, 5 min); 50 µl of this solution was injected into the chromatograph.

### 2.7 Method validation for HPLC

In accordance with the standard guideline ^15^ for method validation, selectivity, LLOQ, carry‐over, linearity, accuracy, precision, dilution integrity, and stability were evaluated.

#### 2.7.1 Selectivity and LLOQ

Methotrexate‐ and IS‐free CSF samples were used to evaluate the selectivity. The lowest concentration on the calibration curve was regarded as the LLOQ. For MTX, interfering peak areas were <20% of the peak area of the LLOQ. For LLOQ samples, the mean accuracy was within ±20% of the nominal value and the coefficient of variation (CV) value did not exceed 20%.

#### 2.7.2 Carry‐over and linearity

The carry‐over effects of MTX and IS were evaluated by testing the response of a blank CSF sample immediately following the highest concentration of the calibration sample. The peak area of the blank CSF sample was <20% of the peak area of the LLOQ sample for MTX and 5% for the IS. The ordinary least squares method was used to fit the peak area ratio vs. the analyte concentration for linearity.

#### 2.7.3 Accuracy and precision

To evaluate the accuracy and precision, five replicates of LLOQ samples and QC samples at three levels were analyzed. The mean accuracy was within ±15% of the nominal values for the QC samples, except for the LLOQ, which was within ±20% of the nominal value. The CV value did not exceed 15% for the QC samples and 20% for the LLOQ samples.

#### 2.7.4 Dilution integrity and stability

To evaluate the dilution integrity for the clinical samples, a 10‐fold dilution of QC samples (at a concentration of 10 µM, exceeding the highest calibration sample) using blank CSF was performed. Five replicates of diluted samples were analyzed. The accuracy and precision of the diluted samples were within ±15% and 15%, respectively. Stability tests were performed by evaluating the accuracy of QC samples at three levels under two conditions (in the CSF matrix, at 23°C for 24 h and −20°C for 24 h). Five replicates of QC samples at three levels were analyzed. The accuracy of QC samples for the stability test was within ±15%.

### 2.8 CLIA

The CLIA was performed on the ARCHITECT i1000SR system (Abbott). The recommended matrices included serum and plasma. The sample volume was 60 µl. The calibration range was 0.040‐1.500 µM. Samples with MTX concentrations >1.500 µM were diluted 20‐fold with the ARCHITECT i1000SR system mechanically. Calibration (ARCHITECT Methotrexate Calibrators, Abbott) and QC samples (ARCHITECT Methotrexate Controls, Abbott) were routinely tested according to the manufacturer's instructions before the analysis of the samples.

#### 2.8.1 Linearity

Linearity of MTX detection in CSF was assessed using samples prepared by the addition of properly diluted stock solution to drug‐free CSF (0.1, 0.2, 0.3, 0.4, 0.5, 0.6, 0.7, 0.8, 0.9, 1.0, 1.1, 1.2, 1.3, 1.4, 1.5, 2.0, 2.5, 3.0, 5.0, 6.0, 7.0, 8.0, 9.0, 10.0, 11.0, 12.0, 13.0, 14.0, and 15.0 µM).

#### 2.8.2 Precision

Four samples with MTX concentrations of 0.070, 0.450, 1.000, and 10.000 µM were prepared by the addition of properly diluted stock solution to drug‐free CSF. To evaluate the precision, five replicates of samples at each level were analyzed.

### 2.9 Application and comparison

A total of 47 CSF samples (including 14 clinical and 33 spiked specimens) were analyzed using the two methods. For HPLC, samples with MTX concentrations >1.50 µM were diluted 10‐fold with the blank CSF. For CLIA, samples with MTX concentrations >1.500 µM were diluted 20‐fold with the ARCHITECT i1000SR system mechanically. A Bland‐Altman plot was used to evaluate the agreement between HPLC and CLIA for the analysis of MTX in human CSF.^16^, ^17^ The equation and the correlation coefficient describing the relationship between the two methods were evaluated using the Passing‐Bablok regression ^18^ and Pearson's product‐moment correlation analysis.

### 2.10 Ethics approval

This study was approved by the ethics review committee of the Faculty of Medicine at the University of Miyazaki, Japan (O‐0638, O‐0676).

### 2.11 Statistical analysis

R v.3.5.1 (www.r‐project.org) was used for statistical analyses. Results with a *p*‐value of <.05 were regarded as statistically significant.

## 3 RESULTS

### 3.1 Method validation for HPLC

#### 3.1.1 LLOQ and selectivity

**Figure 1:**
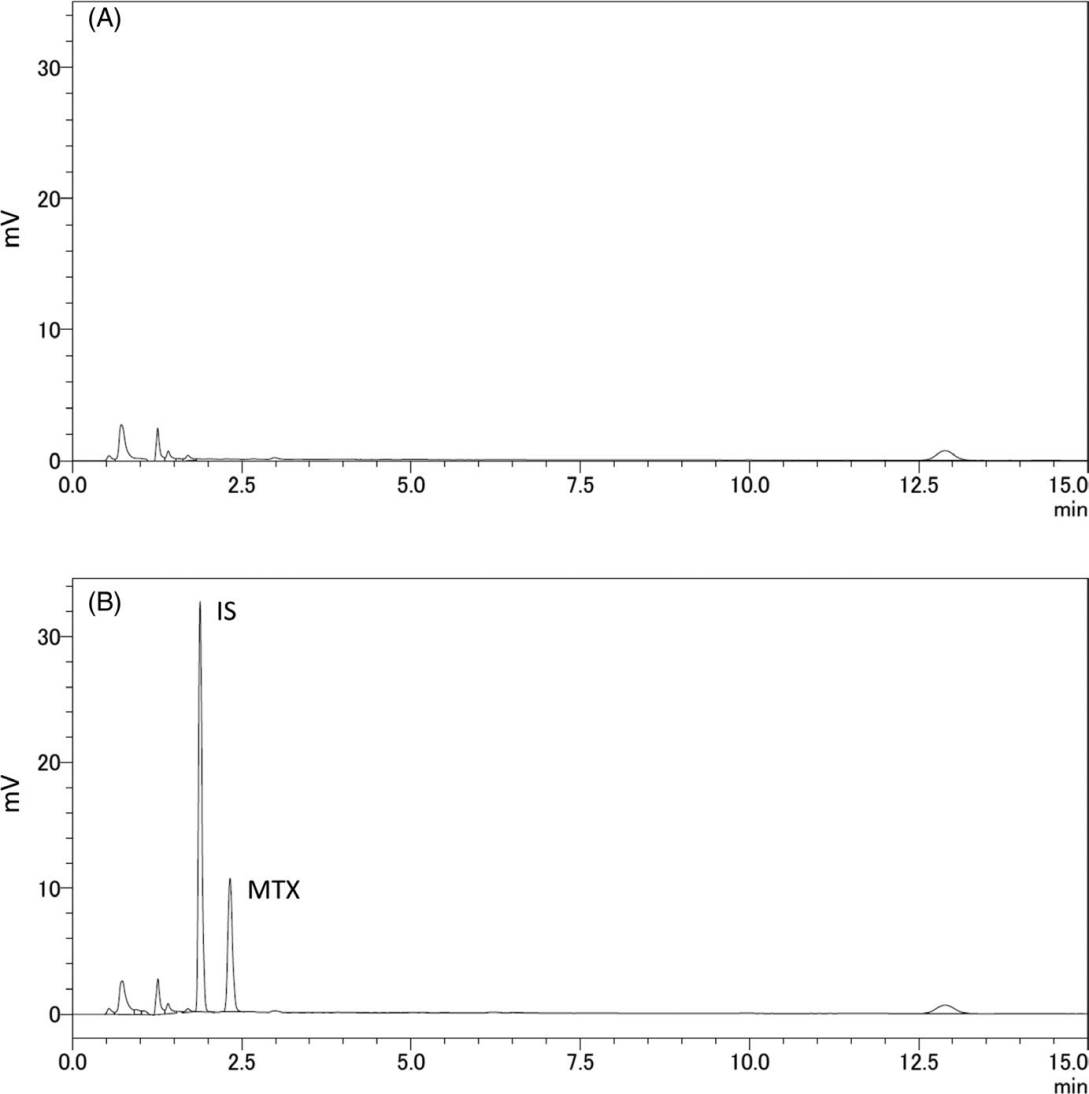
HPLC chromatograms of blank CSF (A); calibration sample of 1.50 µM methotrexate and the IS (B). HPLC conditions: isocratic elution with the mobile phase comprising sodium acetate buffer (50 mM, pH 3.6):acetonitrile (77:13, v/v); UV detector wavelength, 307 nm. HPLC: high‐performance liquid chromatography, CSF: cerebrospinal fluid, MTX: methotrexate, IS: internal standard

The typical chromatograms obtained using the HPLC method are shown in Figure 1. The interfering peaks were not observed in the blank CSF sample (Figure 1A) at the retention times for MTX and IS (Figure 1B). The IS did not affect the measurement of MTX. For LLOQ samples, the mean relative error was 15.8% and the CV was 1.97%.

#### 3.1.2 Carry‐over and linearity

**Figure 2:**
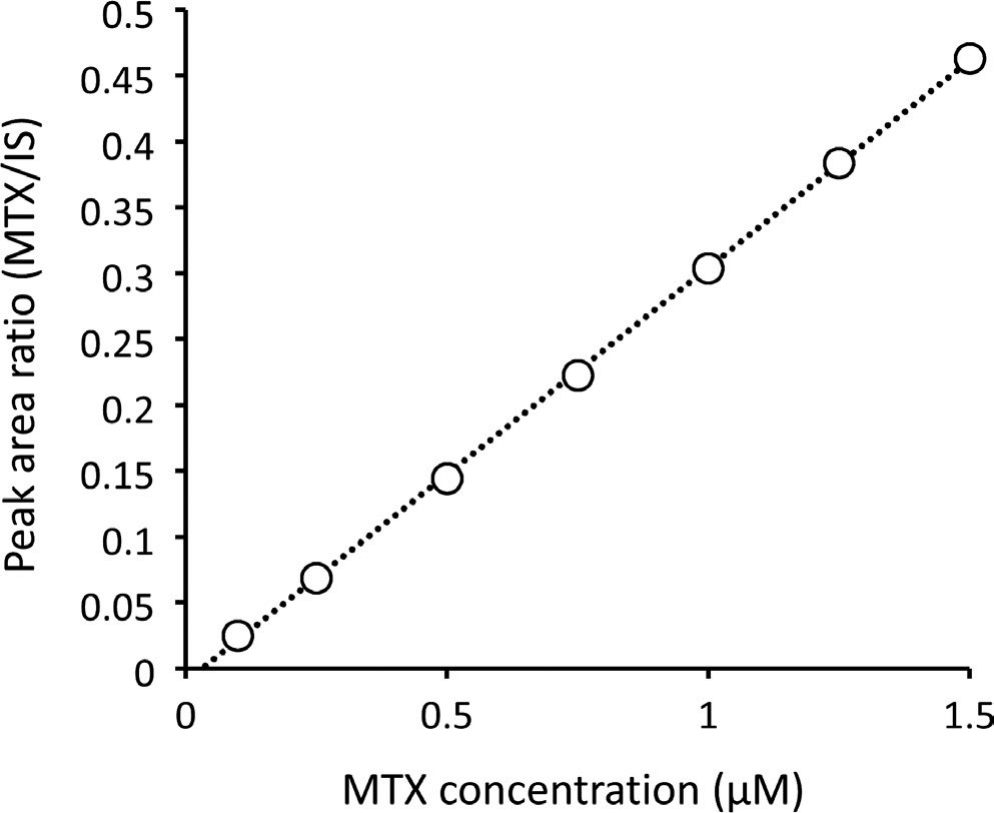
A typical calibration curve. A series of calibration samples was prepared using 0.10, 0.25, 0.50, 0.75, 1.00, 1.25, and 1.50 µM of MTX. MTX: methotrexate

No carry‐over was observed. A typical calibration curve is shown in Figure 2. The linear regression equation was found to be as follows: *y* = 0.3144*x* – 0.0102 (*x*, concentration of MTX; *y*, peak area ratio of MTX to IS, *r* = .999).

#### 3.1.3 Accuracy and precision

The results for the accuracy and precision of the HPLC method are presented in Table 1. For the three concentrations of the QC samples, the mean relative error and CV were < 15%. For LLOQ samples, the mean relative error and the CV were <20%.

**Table 1:** Accuracy and precision of the HPLC method in the measurement of methotrexate concentration in the human cerebrospinal fluid

Concentration (µM)		CV (%)	Relative error (%)
Spiked	Measured (mean ± SD)		
0.10	0.116 ± 0.002	1.97	15.8
0.20	0.199 ± 0.003	1.40	1.2
0.60	0.598 ± 0.007	1.10	0.8
1.20	1.170 ± 0.018	1.56	2.6

Note

N = 5.

Abbreviations: CV, coefficient of variation; SD, standard deviation.

#### 3.1.4 Dilution integrity and stability

For diluted samples, the mean relative error was 4.4% and the CV was 1.88%. The results of the stability tests are shown in Table 2. Because the mean relative error for each condition and level was <15%, MTX was stable in CSF under all the tested conditions at concentrations of 0.20, 0.60, and 1.20 µM.

**Table 2:** Stability of methotrexate in human cerebrospinal fluid under tested conditions

	Concentration (µM)		CV (%)	Relative error (%)
	Spiked	Measured (mean ± SD)		
24 h, 23°C	0.20	0.227 ± 0.004	1.91	13.3
	0.60	0.658 ± 0.015	2.27	9.7
	1.20	1.291 ± 0.036	2.80	7.6
24 h, −20°C	0.20	0.221 ± 0.004	1.74	10.4
	0.60	0.652 ± 0.008	1.18	8.7
	1.20	1.269 ± 0.028	2.24	5.7

Note

N = 5.

Abbreviations: CV, coefficient of variation; SD, standard deviation.

### 3.2 Method evaluation for CLIA

#### 3.2.1 Linearity

The linearity of MTX detection in CSF by CLIA was excellent in the verified concentration range (0.1‐15.0 µM); the correlation coefficient (*r*) was 0.997.

#### 3.2.2 Precision

For the four samples with MTX concentrations of 0.070, 0.450, 1.000, and 10.000 µM, the CVs of five replicates were 3.14%, 2.77%, 1.79%, and 2.98%, respectively.

### 3.3 Method comparison

**Figure 3:**
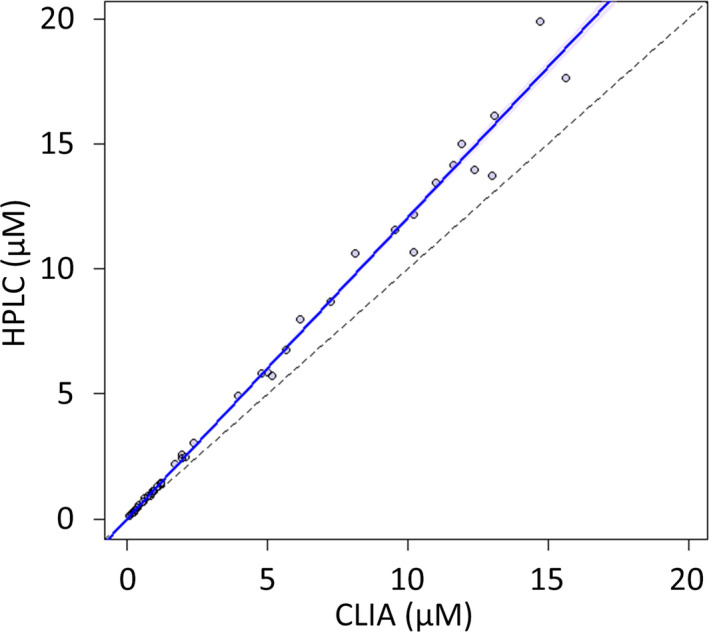
Passing‐Bablok regression of the HPLC method and CLIA method for the analysis of methotrexate in CSF. HPLC: high‐performance liquid chromatography, CLIA: chemiluminescence immunoassay, MTX: methotrexate, CSF: cerebrospinal fluid

**Figure 4:**
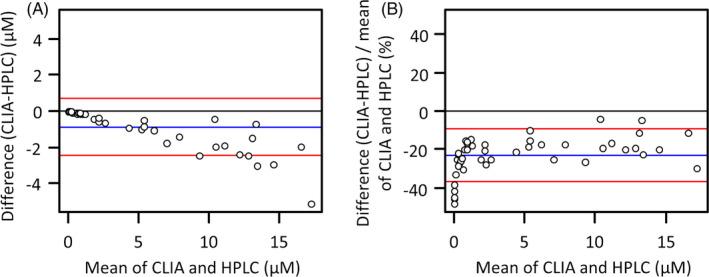
Bland‐Altman plot of the HPLC method and CLIA method for the analysis of methotrexate in CSF. (A) Actual difference in the concentration measured using the CLIA method and using the HPLC method. (B) Percent difference in the concentration measured using the CLIA method and using the HPLC method. The blue line shows the average, and the red lines show the 95% confidence interval. HPLC: high‐performance liquid chromatography, CLIA: chemiluminescence immunoassay, MTX: methotrexate, CSF: cerebrospinal fluid

The ranges of MTX measured using the CLIA and HPLC methods were 0.071‐15.61 µM and 0.108‐19.89 µM, respectively, including the concentrations measured in clinical and spiked specimens. Based on the Passing‐Bablok regression (Figure 3), the relationship between the concentrations determined using the two methods was as follows: *HPLC* = 1.205 × *CLIA* – 0.024, r = 0.995. From the Bland‐Altman plot, a proportional error was observed between the results obtained using the two methods (r = −0.878, *p* < .01) (Figure 4A). In addition, evaluation using a re‐plotting after the incorporation of the percent difference showed that the average magnitude of the proportional error was −23.0% (95% confidence interval: −36.9% to −9.1%) (Figure 4B).

## 4 DISCUSSION

In this study, we established an HPLC method for measuring the concentration of MTX in the CSF and successfully verified the concentration of MTX in the CSF measured using the CLIA method. The monitoring of the MTX concentration in the CSF is important for preventing its toxicity to the central nervous system and evaluating the therapeutic effects. The LLOQ in the HPLC method was determined to be 0.10 µM, which is considered sufficiently sensitive for the toxicity standard of MTX (1 µM).^5^ It has been reported that the concentration of MTX in the CSF, 24 h after the intraventricular administration of MTX, does not exceed 5 µM.^19^ Therefore, based on the detection range and the accuracy of the quantitative value of the diluted sample, the concentration of MTX in most CSF samples can be accurately quantified. However, this assumption cannot be generalized because some cases may present an irregular delay in the excretion of MTX from the ventricular CSF. In the CSF, MTX was sufficiently stable in the environment of routine sample transport, and the analytical results for clinical samples were considered reliable.

Because immunoassays are based on antigen‐antibody interactions, cross‐reactivity is an important issue. Drugs that are analyzed after in vivo administration undergo minute structural changes because of chemical reactions, such as metabolism, and can be converted into non‐analyte targets. If a drug is to be analyzed, these non‐analyte targets may be detected. Furthermore, when the sample to be analyzed is directly supplied to the reaction system, there is a possibility that the reaction is affected by the sample components. Although immunoassays are less versatile than separation analyses methods, they are very much applicable for clinical use, such as in commercial reagent kits. In this study, we measured the concentrations of MTX in different matrix samples using an immunoassay for which the intended matrix is serum or plasma. The concentration of MTX in the CSF sample measured using the CLIA method was slightly different from that measured using the HPLC method. The concentration determined using the HPLC method developed for the quantification of the MTX concentration in the CSF can be considered the actual value. We found that the concentration of MTX in the CSF could not be accurately measured by the CLIA method intended for serum or plasma matrices. Because of the cross‐reactivity, it is easily considered that the results obtained using the CLIA method would be higher than those obtained using the HPLC method. In addition, there is a concern that protein in the sample could influence drug quantification by the immune reaction. The protein binding rate of MTX in blood is about 50%.^20^ The documentation for the reagent kit for the CLIA method states that the measured value may be low because of high albumin content in the sample. However, the amount of protein in CSF is significantly lower than that in blood because of the presence of the blood‐CSF barrier. Hence, we believe MTX quantification in the CSF sample is not easily affected by protein. However, the results obtained using the CLIA method were actually lower than those obtained using the HPLC method. In immunoassays, the pH and ionic concentration of the sample affects the reactivity.^21^, ^22^, ^23^ These factors may have affected the results obtained in this study. In contrast, the differences between the values obtained using the CLIA and HPLC methods were significant proportional errors. Therefore, we believe that it is possible to correct the measured value obtained using the CLIA method with the equation (*HPLC* = 1.205 × *CLIA* – 0.024) obtained from the Passing‐Bablok regression, and this corrected value can be used in clinical practice. However, correction with this equation can only be performed in the concentration range (0.1‐15.0 µM) in which the linearity of the CLIA method was verified, and the correction accuracy should be verified in the future. Furthermore, this error in the determined values can be solved by constructing a CSF‐based calibration curve for the immunoassay. However, in a fully automatic analyzer, such as the ARCHITECT i1000SR system, the calibration curve is saved in the control system. For daily clinical practice, it is extremely useful to have an environment in which the concentration of MTX in serum, plasma, and CSF can be measured with one calibration curve (serum and plasma bases with higher measurement frequency, dedicated commercial kit). Nevertheless, it was shown that high and low MTX concentrations in CSF can be evaluated by the CLIA method. The adaptability of the correction value to the clinical criteria of MTX concentration in CSF requires further study, but irregular intraventricular drug clearance can be sufficiently evaluated. Moreover, the ARCHITECT i1000SR system can analyze more than 30 samples per hour, which is highly valuable for clinical applications requiring a rapid, simple, and convenient method.

There are some limitations to our study. First, the number of clinical specimens used in the comparison of the two methods was few. Although a CSF sample spiked with MTX was used to ensure a wide range of comparative concentrations, data that are completely consistent with clinical samples might not have been obtained. Second, the accuracy of the regression equation used for correcting the values measured using the CLIA method could not be verified using a clinical sample different from the samples used to create the regression equation. This was because of the scarcity of specimens.

In this study, we verified the accuracy of measuring MTX concentrations in the CSF using the CLIA method and deduced an equation that can correct the values with respect to the actual ones determined using the HPLC method. The evaluation of the existing immunoassays other than the CLIA method in the same manner may lead to further adoption of the measurement of the MTX concentration in the CSF in routine clinical practice. We believe such evaluations will contribute to providing safer and more personalized treatment.

## CONFLICT OF INTEREST

The authors declare that they have no conflict of interest.

## Data Availability

The data that support the findings of this study are available from the corresponding author, NY, upon reasonable request.
